# Hypoxia Constructing the Prognostic Model of Colorectal Adenocarcinoma and Related to the Immune Microenvironment

**DOI:** 10.3389/fcell.2021.665364

**Published:** 2021-04-20

**Authors:** Yuanyuan Zhang, Feng Yang, Xiaohong Peng, Xiaoyu Li, Na Luo, Wenjun Zhu, Min Fu, Qianxia Li, Guangyuan Hu

**Affiliations:** Department of Oncology, Tongji Hospital, Tongji Medical College, Huazhong University of Science and Technology, Wuhan, China

**Keywords:** bioinformatic analysis, hypoxia, colorectal adenocarcinoma, survival, prognosis

## Abstract

**Background:** Hypoxia is a common phenomenon in solid tumors, which plays an important role in tumor proliferation, apoptosis, angiogenesis, invasion and metastasis, energy metabolism and chemoradiotherapy resistance. However, comprehensive analysis of hypoxia markers in colorectal adenocarcinoma (COAD) is still lacking. And there is a need for mechanism exploration and clinical application.

**Methods:** The gene expression, mutation and clinical data of COAD were downloaded from The Cancer Genome Atlas (TCGA) and the Gene Expression Omnibus (GEO) databases, respectively. Tumor samples from TCGA were randomly divided into the training and internal validation groups, while tumor samples from GEO were used as the external validation group. Univariate COX—LASSO—multivariate COX method was applied to construct the prognostic model. We clustered all TCGA tumor samples into high, medium and low hypoxia groups, evaluated the correlation between hypoxia degree and immunoactivity, and explored the combined effect of mutation for common target genes and model riskscore on survival in COAD patients. Finally, we developed a dynamic nomograph App online for direct clinical application and carried out multiple validations of the prognostic model.

**Results:** Our hypoxia-related prognostic model for COAD patients is accurate and has been successfully validated internally and externally. Single Sample Gene Set Enrichment Analysis (ssGSEA) and Gene Set Enrichment Analysis (GSEA) results suggest that for COAD patients with higher hypoxia, the stronger the associated immunosuppressive activity, providing a possible mechanism for the lower survival rate. Finally, the dynamic nomograph App online enhances the clinical translational significance of the study.

**Conclusion:** In this study, an accurate prognostic model for COAD patients was established and validated. In addition, our innovative findings include correlations between hypoxia levels and immune activity, as well as an in-depth exploration of common target gene mutations.

## Introduction

COAD is the most common pathological type of colon cancer, with melena, iron deficiency anemia, abdominal pain and changes in defecation habits as the main symptoms (Siegel et al., 2019). The 5-year survival rate of COAD is related to the stage of disease development. The overall 5-year survival rate is 64%, while the 5-year survival rate for patients with distal metastasis drops to 14% (Siegel et al., 2020). Cancer cells of advanced COAD have a high degree of proliferation and complete cure is difficult to achieve at this time, so chemotherapy is mainly used to prolong the survival period. Therefore, it is necessary to construct prognostic signatures to stratify COAD patients for risk and implement early intervention.

The metabolism of tumor cells is active, so hypoxia is often present in the center. For tumor cells, hypoxia promotes angiogenesis and remodeling by inducing HIF expression enhancement, and is a marker of tumor proliferation, metastasis, and recurrence (Muz et al., 2015). Potential mechanisms include altered gene expression, oncogene activation, antioncogene inactivation, decreased genomic stability and clonal selection (Emami Nejad et al., 2021). Therefore, inhibiting the production of HIF has become an important research direction of tumor therapy. In addition, hypoxia can lead to immunosuppression in solid tumors, making it a potential target for improving immunotherapy (Wang et al., 2021). At present, more hypoxia inducible factor inhibitors are under development and have broad application prospects (Takahashi et al., 2019; Zhang et al., 2019; Marti-Diaz et al., 2020; Zhou et al., 2020).

Relevant data have shown that hypoxia enhances HIF-1 and VEGF expression in COAD tissue, thereby promoting angiogenesis and tumor progression (Wang et al., 2010). For COAD, hypoxia promotes epithelial-mesenchymal transformation (EMT), which leads to cytoskeleton recombination and ultimately to further migration and invasion of tumor cells (Choi et al., 2017). Small molecule vascular endothelial growth factor receptor inhibitors improved the survival of metastatic COAD patients with high serum LDH (a potential marker of hypoxia) in a randomized, controlled phase III clinical trial (Hecht et al., 2011). Tocotrienol, an anti-angiogenic agent, inhibits the production of angiogenic factor by inhibiting HIF-1α, which significantly reduces tumor proliferation (Shibata et al., 2008). More similar drugs are being developed that are expected to help improve the survival of COAD patients (especially with advanced metastasis) (Jia et al., 2016).

We downloaded datasets from multiple public databases and performed a wide variety of bioinformatics analyses. Firstly, univariate COX—LASSO—multivariate COX regression was used to construct the hypoxia-related prognosis model, and dynamic nomograph was plotted. Secondly, the correlation between hypoxia and immunity was compared by combining ssGSEA and clustering algorithm. Next, differences in gene mutation between the high and low risk groups were revealed. Finally, several online databases were applied for validation.

## Materials and Methods

### Data Acquisition and Differential Analysis

We downloaded the TCGA-COAD gene expression dataset and sample information file from the TCGA website (Cancer Genome Atlas Research Network et al., 2013). We also retrieved the COAD probe matrix file (GSE38832_series_matrix) and the platform file (GPL570-55999) from the GEO database (Barrett et al., 2005). The hypoxia-related genes were obtained from the GSEA website (Subramanian et al., 2005). In addition, COAD mutation data (TCGA.COAD.varscan.8177ce4f-02d8-4d75-a0d6-1c5450ee08b0.DR-10.0.somatic) was also downloaded from the TCGA website. All samples were from newly diagnosed patients. All data has been cleaned and calibrated by using limma (Ritchie et al., 2015) and sva (Leek et al., 2012) packages, respectively.

Firstly, we used GSEA software to validate whether the hypoxia-related enrichment terms were upregulated in the tumor group. Secondly, we adopted Wilcox test to analyze the differential hypoxia genes between normal and tumor groups (FDR < 0.05 and | logFC | > 0.5), and drew the volcano plot and differential heat map.

Finally, we carried differential hypoxia genes into the Weighted Gene Co-expression Network Analysis (WGCNA) analysis (Langfelder and Horvath, 2008) to find disease-related modules (the modules that differed most significantly between normal and tumor samples). Subsequently, we inputted the genes in these modules into the Search Tool for the Retrieval of Interacting Genes (STRING) database (Szklarczyk et al., 2017) and Cytoscape software (Su et al., 2014) and obtained the protein-protein interaction (PPI) network core through multiple filtering of CytoNCA plug-in (Tang et al., 2015).

### Construction of Prognostic Model and Survival Analysis

TCGA samples were randomly divided into the training and internal validation groups with caret package (Kuhn, 2008), while GEO samples were used as the external validation group. In the training group, univariate COX analysis found survival-related hypoxia genes (*P* < 0.01), and least absolute shrinkage and selection operator (LASSO) algorithm (Tibshirani, 1997) removed highly correlated genes to prevent model overfitting. At last, stepwise multivariate COX regression analysis constructed the prognostic model successfully.

With the median riskScore of the training group as the threshold, patients in the training and validation groups were divided into the high and low risk groups. Survival curves and receiver operating characteristic (ROC) curves of the training and validation groups were plotted, respectively. Meanwhile, univariate and multivariate prognostic analyses (*P* < 0.05) were conducted for the training group to determine whether the riskScore obtained from the model could be an independent prognostic factor.

On the basis of the prognostic model, we analyzed the correlation between each model gene and tumor stage, immune subtype, stem cell index and tumor microenvironmental parameters.

### ssGSEA Analysis and Mutation Data

We performed ssGSEA analysis by using the GSVA package (Hanzelmann et al., 2013) to obtain the immunoactivity of 29 immune-related gene sets in the TCGA-COAD samples. According to the expression level of survival-related hypoxia genes (*P* < 0.01), TCGA-COAD samples were clustered to generate high, medium and low hypoxia groups. And we drew the heat map and violin plot of tumor microenvironment and the survival curve of three hypoxia groups. The org.Hs.eg.db R software package was used for GSEA enrichment analysis, and five Gene Ontology (GO) terms and Kyoto Encyclopedia of Genes and Genomes (KEGG) pathways (*P* < 0.05) were plotted in the high hypoxia group (compared to the low hypoxia group).

The maftools software package (Mayakonda et al., 2018) visualized the TCGA-COAD mutation data and drew the waterfall plots of high and low risk groups. Finally, we compared the effect of common target gene mutations on patients’ survival in high and low risk groups.

### Dynamic Nomogram and Validations

In order to improve the practicability of our prognostic model, we adopted the DynNom package to develop a dynamic nomograph App online, which can accurately predict the survival rate of patients. In the end, we applied the PROGgeneV2 database (Goswami and Nakshatri, 2014) to validate the validity of the model again. The Human Protein Atlas (HPA) database (Uhlen et al., 2005) showed the distribution of model genes in the tissue.

### Statistical Analyses

All statistical analyses were performed using R software (version 3.6.3) unless otherwise stated. Univariate and multivariate COX regression analyses were used to investigate the prognostic value of COAD-related hypoxia indicators. For all statistical results, *P* < 0.05 was considered statistically significant.

## Results

### Hypoxia Genes Differentially Expressed Between Normal and Tumor Samples

**FIGURE 1:**
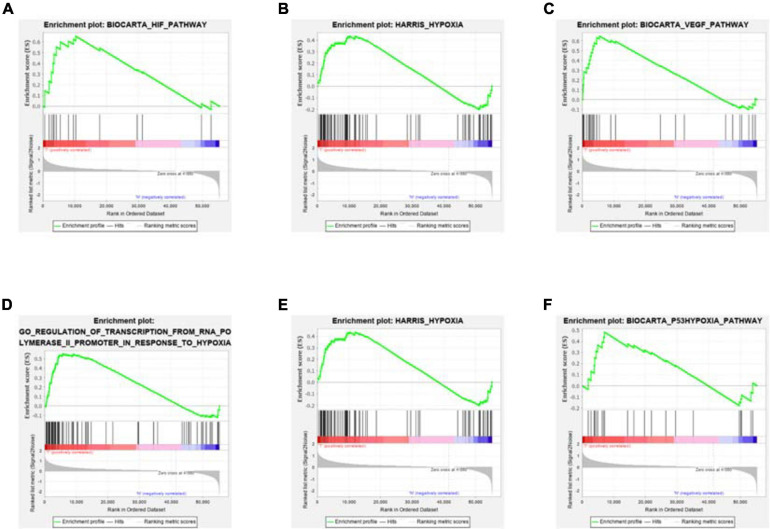
GSEA enrichment results. Compared with the normal samples, most hypoxia-related enrichment terms are significantly upregulated in COAD. Among them, for **(A–D)** figures, *P* < 0.05; for **(E,F)** figures, *P* > 0.05.

**FIGURE 2:**
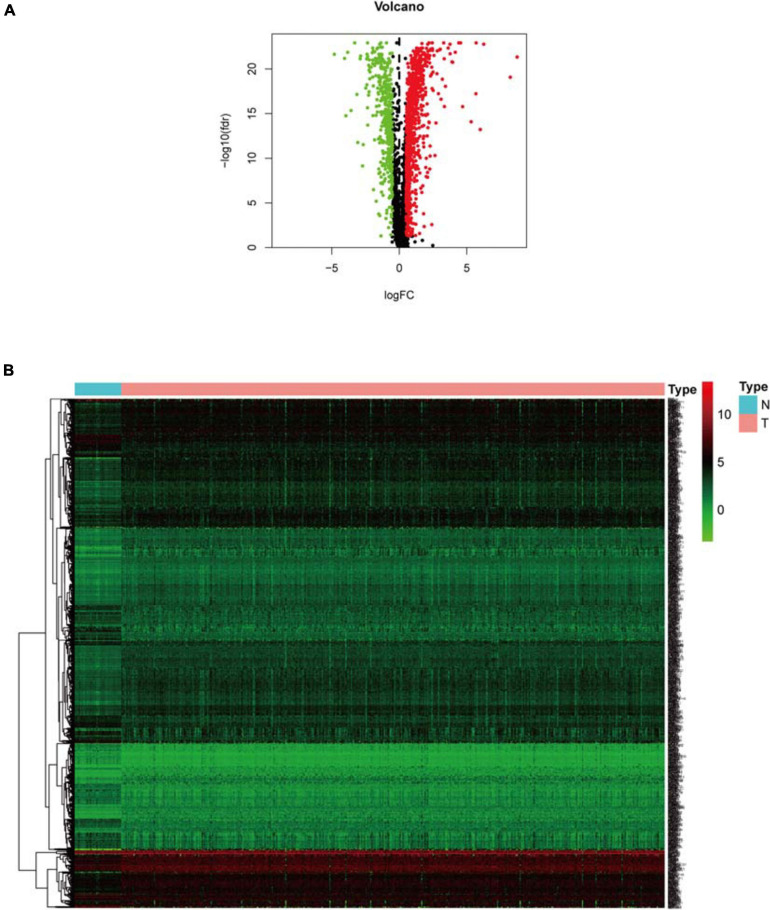
Expression analysis of differential hypoxia genes. **(A)** Volcano plot. Green dots and red dots represent down-regulated and up-regulated genes in COAD samples, respectively. **(B)** Heat map. Green and red correspond to genes with low and high expression levels, respectively.

Clinical information of TCGA-COAD is presented in Table 1 and Supplementary File 1. The operation results of GSEA software shows that, compared to the normal group, most hypoxia-related enrichment terms are significantly upregulated in the tumor group (Figure 1), suggesting that hypoxia-related genes and pathways may play an important role in the occurrence and development of COAD. A total of 1,346 differential hypoxia genes between the normal group and the tumor group were analyzed by Wilcox test (Supplementary File 2), which are visualized in the volcano plot and differential heat map (Figure 2). The volcano plot displays the overall differential distribution of hypoxia genes expression level between normal and tumor samples. The heat map shows changes in the expression of each hypoxia gene in normal and tumor samples.

**TABLE 1:** Clinical characteristics of COAD patients in the TCGA database.

**Characteristics**	**Total patients (*N* = 459)**	
	**No**	**%**
**Age (y)**		
<65	174	37.91
≥65	285	62.09
**Pathologic M**		
M0	337	73.42
M1	65	14.16
MX	50	10.89
Unknown	7	1.53
**Pathologic N**		
N0	270	58.82
N1	106	23.09
N2	83	18.08
**Pathologic T**		
Tis	1	0.22
T1	11	2.40
T2	78	16.99
T3	313	68.19
T4	56	12.20
**Gender**		
Female	216	47.06
Male	243	52.94
**Tumor stage**		
Stage I	76	16.56
Stage II	178	38.78
Stage III	129	28.10
Stage IV	65	14.16
Unknown	11	2.40
**Vital status**		
Alive	367	79.96
Dead	92	20.04

**FIGURE 3:**
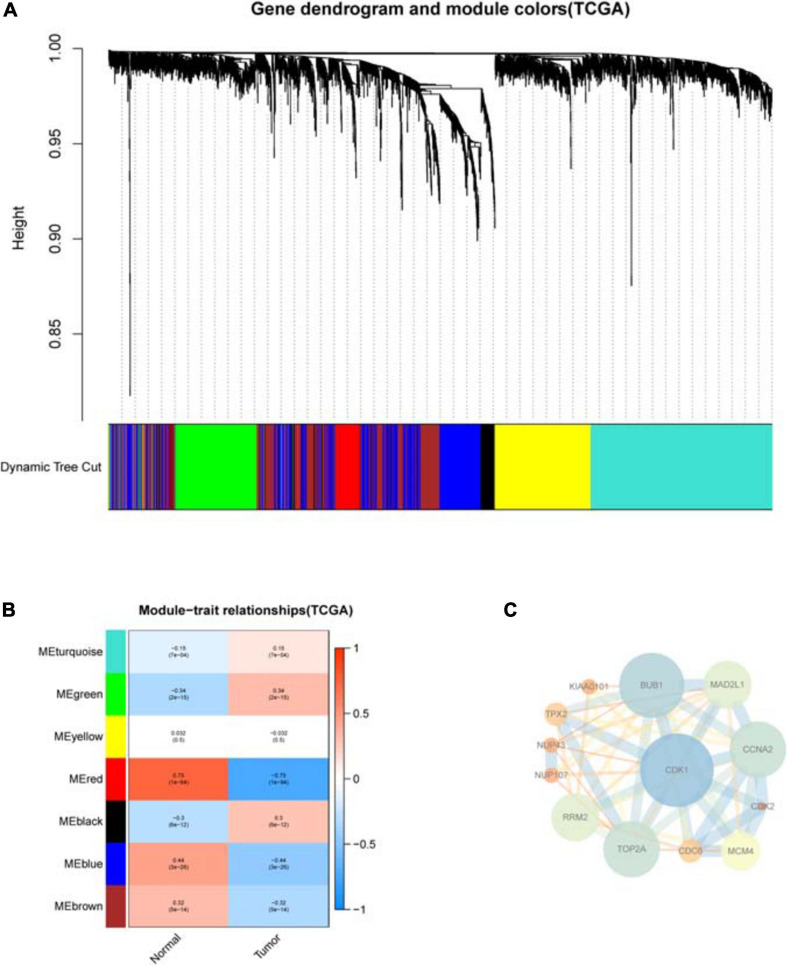
WGCNA analysis of differential hypoxia genes in COAD. **(A)** Cluster tree of disease-related modules. Each module has a different color. **(B)** Heat map of the correlation between modules and sample type. Each row corresponds to a module, and each column corresponds to cancer or normal sample. Each square contains a corresponding correlation and *P*-value. Red represents positive correlation, and blue represents negative correlation. As the color gets darker, the correlation increases. **(C)** The PPI network core of genes in disease-related modules. Nodes represent genes and edges represent interactions between nodes. For nodes, low degrees correspond to small sizes and bright colors, and high degrees correspond to large sizes and dark colors; for edges, low combined_scores correspond to small sizes and bright colors, and high combined_scores correspond to large sizes and dark colors.

WGCNA analysis identifies disease-related modules (Figures 3A,B), among which the MEgreen module has the most significant positive correlation with COAD, while the MEred and MEblue modules have the most significant negative correlation with COAD. The PPI network includes genes from the MEgreen, MEred, and MEblue modules, and the core of the PPI network is finally obtained after repeated filtering by CytoNCA plug-in (Figure 3C). It can be seen that CDK1 gene is located in the central node, which interacts most closely with other differential hypoxia genes.

### The Constructed Prognostic Model and Survival Analysis

**FIGURE 4:**
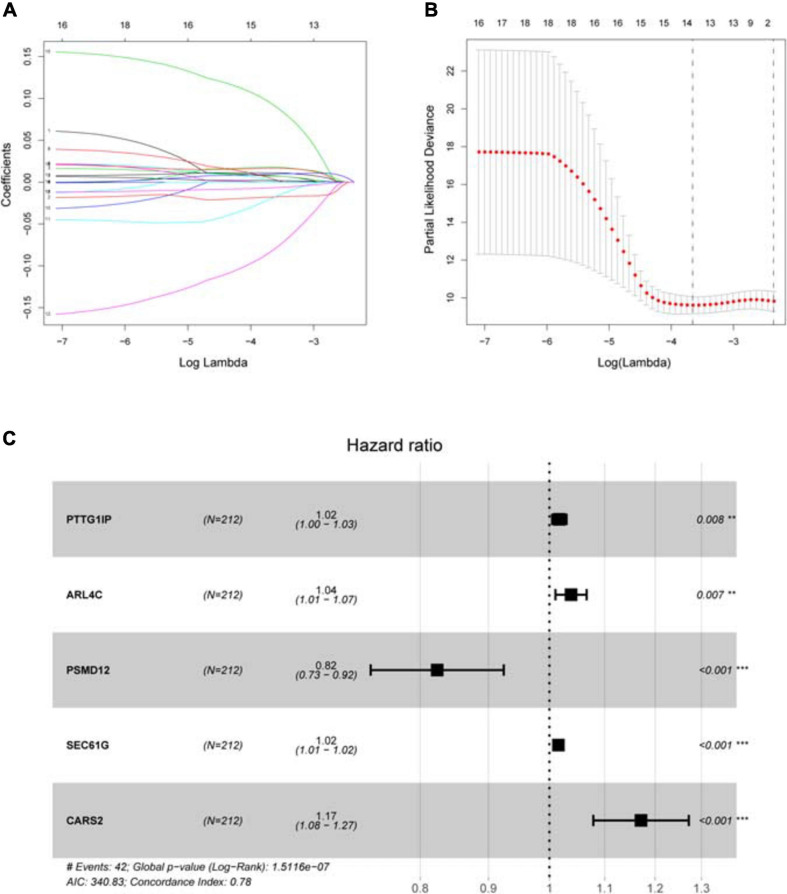
Establishing a hypoxia prognostic model by LASSO regression analysis. **(A)** LASSO coefficient plot of 18 candidate survival-related genes in the training group. The hypoxia genes with non-zero coefficient are identified through the optimal Log Lambda value for the subsequent model construction. **(B)** The best Log Lambda value (corresponding to the minimum cross validation error point) is selected for the training group in the LASSO model, with a vertical dotted line drawn here. **(C)** The forest map visually shows HR values and 95% confidence intervals for all model genes. HR < 1 indicates that this gene is a low-risk gene; otherwise, it is a high-risk gene. *P* < 0.05 indicates that this gene is an independent prognostic factor.

Sample information of the TCGA training group (Supplementary File 3), TCGA internal validation group (Supplementary File 4) and GEO external validation group (Supplementary File 5) has been uploaded. Univariate COX analysis (Supplementary File 6) identifies 18 survival-related hypoxia genes. After further implementation of LASSO algorithm (Figures 4A,B) and stepwise multivariate COX regression analysis, five hypoxia genes are finally involved in the construction of the prognostic model, that is each patient’s riskScore = 0.017^∗^PTTG1IP + 0.037^∗^ARL4C + (−0.194)^∗^PSMD12 + 0.015^∗^SEC61G + 0.158^∗^CARS2 (Figure 4C and Table 2). All model genes are independent prognostic factors. Among them, PSMD12 is a low risk gene, and the other genes are high risk genes.

**TABLE 2:** Multivariate COX regression analysis results of model genes.

**Id**	**Coef**	**HR**	**HR.95L**	**HR.95H**	***P*-value**
PTTG1IP	0.017019790	1.017165451	1.004539285	1.029950318	0.007571027
ARL4C	0.037295728	1.037999941	1.010386893	1.066367632	0.006705666
PSMD12	−0.193696901	0.823907592	0.734526557	0.924164979	0.000946275
SEC61G	0.015449601	1.015569563	1.007437821	1.023766943	0.000165501
CARS2	0.158093430	1.171275622	1.078687084	1.271811447	0.000168059

**FIGURE 5:**
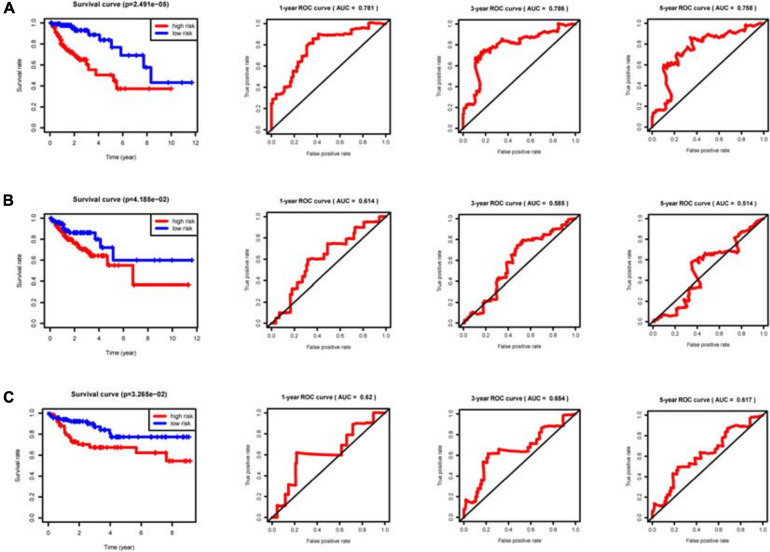
Clinical prognostic model evaluation. The riskScore of the clinical prognostic model can predict survival rate according to Kaplan–Meier (KM) survival analyses for the high and low risk groups. The ROC curve is used to evaluate the prediction efficiency of the prognostic model and validate the prognostic value of the model. **(A)** Training group. **(B)** Internal validation group. **(C)** External validation group GSE38832.

**FIGURE 6:**
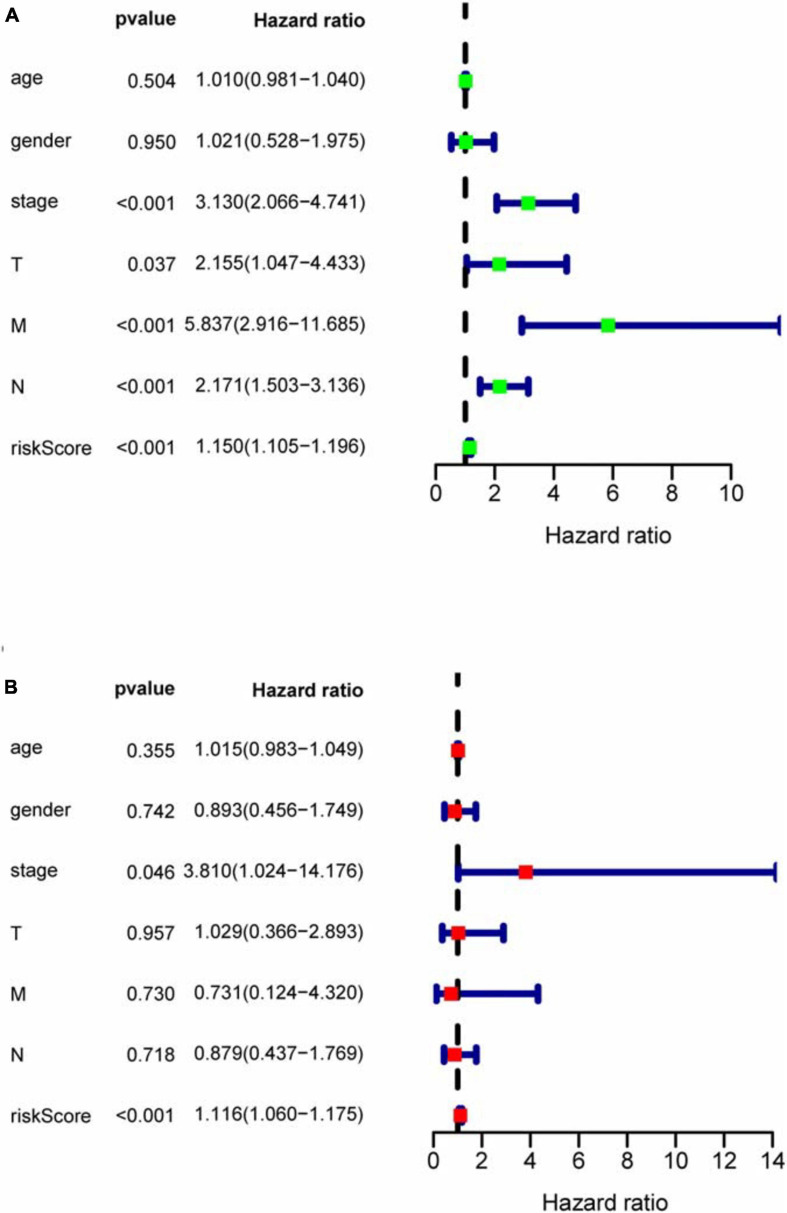
COX regression analysis for assessing the independent prognostic value of riskScore. **(A)** Univariate regression analysis. **(B)** Multivariate regression analysis. In univariate and multivariate independent prognostic analyses, if both *P*-values of riskScore are less than 0.05, riskScore could become an independent prognostic factor.

With the median riskScore of the training group as the cut-off value, patients of the training group, internal validation group and external validation group have been divided into the high and low risk groups, respectively (Supplementary Files 7–9). In the training, internal validation and external validation groups, the survival rate is significantly higher in the low risk group than in the high risk group. Based on the area under the curve (AUC) value of ROC curve, it can be judged that the prognostic model we constructed has good accuracy (Figure 5). Univariate and multivariate prognostic analyses demonstrate that the riskScore of the model could be an independent prognostic factor (Figure 6).

After the prognostic model was established, we analyzed the correlation between each model gene and tumor stage, immune subtype, stem cell index and tumor microenvironmental parameters. It can be seen that PTTG1IP, ARL4C, PSMD12, and CARS2 are significantly associated with tumor stage (Supplementary File 10). The expression levels of ARL4C, PSMD12, and CARS2 are significantly different in various immune subtypes (Supplementary File 11). PTTG1IP and ARL4C are negatively correlated with stem cell index and positively correlated with microenvironment immune cell score. The opposite is true for PSMD12, SEC61G, and CARS2 (Supplementary File 12).

### ssGSEA Analysis and Mutation Data Visualization

**FIGURE 7:**
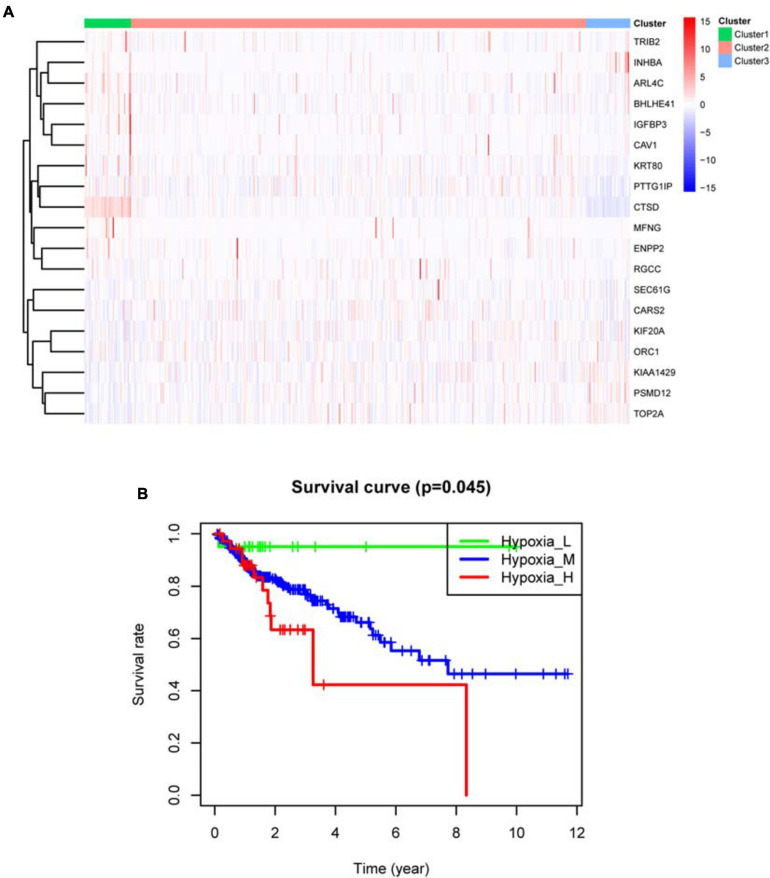
TCGA-COAD sample clustering results. **(A)** Clustering heat map. In the center of the subfigure, high expression of the hypoxia gene is shown in red and low expression of the hypoxia gene is shown in blue. Cluster 1 is mainly red, representing high hypoxia group. Cluster 2 has red and blue, representing medium hypoxia group. Cluster 3 is mainly blue, representing low hypoxia group. **(B)** Kaplan–Meier survival curve. *P* < 0.05 indicates that there is a difference in survival among the three groups.

**FIGURE 8:**
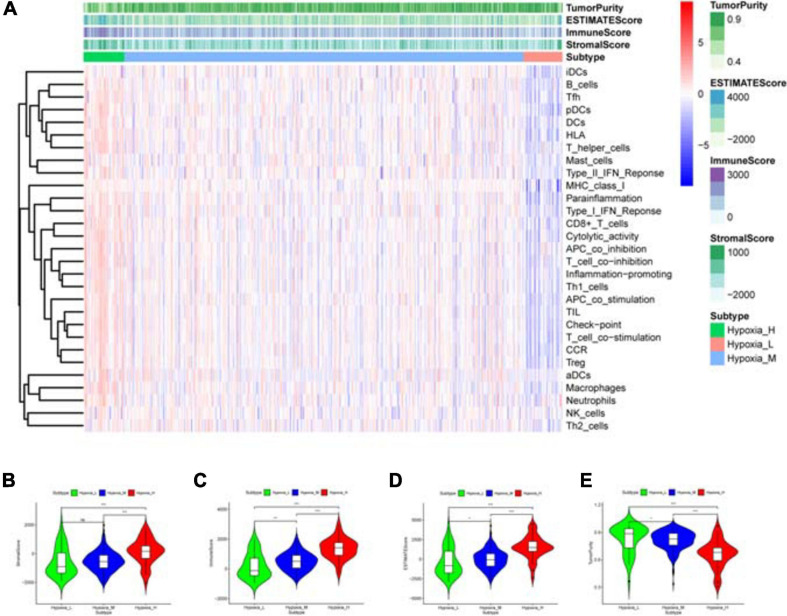
Immune status analysis of tumor microenvironment. **(A)** Heat map. The abscissa represents the sample name and the ordinate represents the immune gene set. The upper part is tumor microenvironment scores, in which StromalScore, ImmuneScore and ESTIMATEScore decrease with the decrease of hypoxia degree, suggesting that the content of corresponding cells decrease. Tumor purity is opposite to them. **(B–E)** Violin plot. The correlation between the hypoxia degree and each tumor microenvironmental parameter is statistically analyzed. ***P < 0.001, **P < 0.01, *P < 0.05, ^*ns*^*P* > 0.05.

**FIGURE 9:**
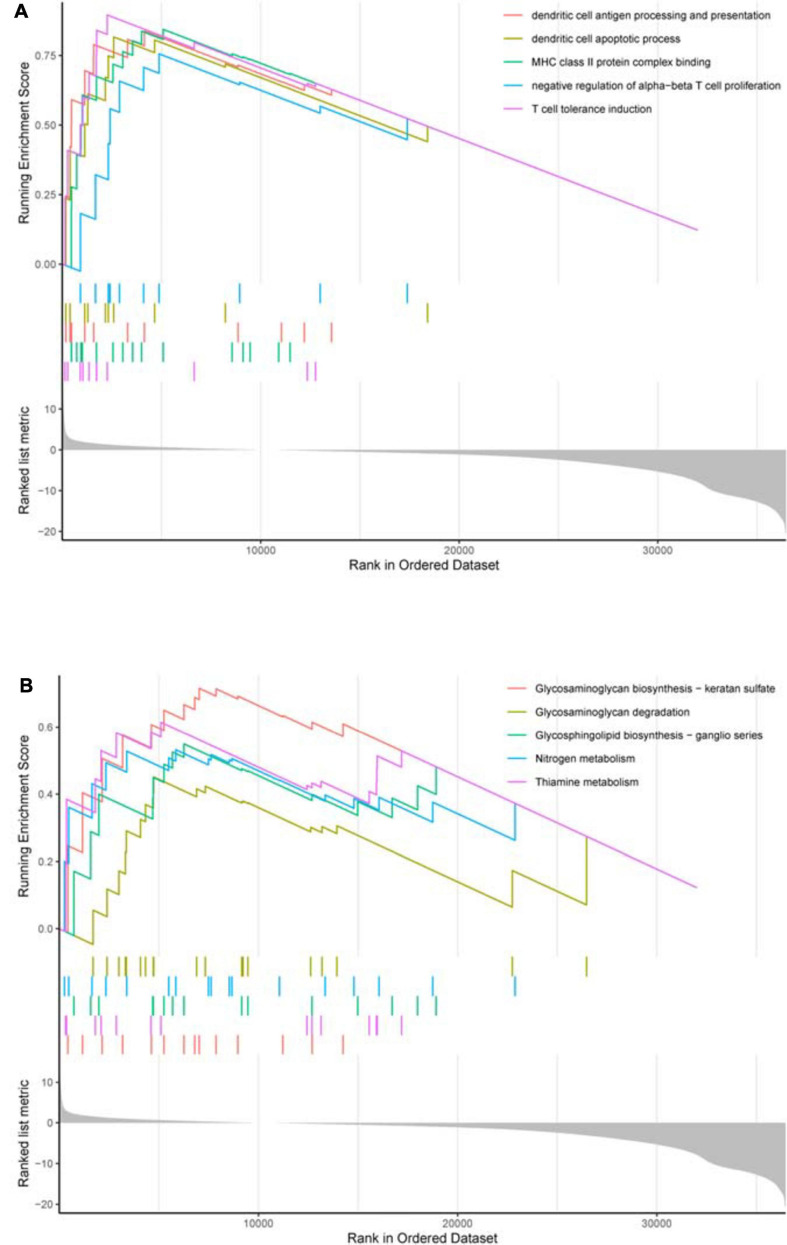
GSEA enrichment analysis. **(A)** GO enrichment result. In the upper section, the highest score of each GO term is the GO enrichment score. These GO terms are significantly enriched in the high hypoxia group. **(B)** KEGG enrichment result. In the upper section, the highest score of each KEGG pathway is the KEGG enrichment score. These KEGG pathways are significantly enriched in the high hypoxia group.

Based on survival-related hypoxia genes, TCGA-COAD samples were clustered to generate high, medium and low hypoxia groups (Figure 7A and Supplementary File 13). The survival curve shows that there is a significant difference in survival rates between the three hypoxia groups (with the higher the degree of hypoxia, the lower the survival rate) (Figure 7B). The heat map (Figure 8A) and violin plot (Figures 8B–E) of tumor microenvironment reflect a significant rule, that is, hypoxia degree is positively correlated with stromalscore, immunoscore and estimatscore, and negatively correlated with tumor purity. GSEA enrichment analysis was conducted between the high and low hypoxia groups to obtain 5 GO terms (Figure 9A) and KEGG pathways (Figure 9B) significantly enriched in the high hypoxia group. These GO terms and KEGG pathways are mainly related to immunity and metabolism.

**FIGURE 10:**
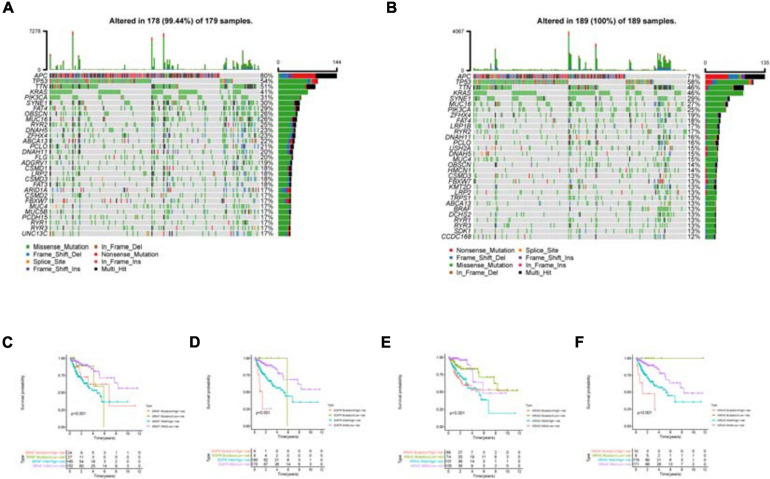
Mutation analysis of the high and low risk groups. **(A)** is the mutation waterfall plot of the low risk group. **(B)** Is the mutation waterfall plot of the high risk group. The ordinate is the gene name and the abscissa is the sample name. Different color represents different mutation type. **(C–F)** Effect of common target gene mutation on patients’ survival in the high and low risk groups.

As shown in the mutation waterfall plot of the high and low risk groups (Figures 10A,B), the genes with the highest mutation frequency in the two groups are APC, TP53 and TTN. Among them, APC mutations are mainly Nonsense Mutation and Multi Hit, while TP53 and TTN mutations are mainly Missense Mutation. By comparing the effects of common target gene mutations on the patients’ survival of the high and low risk groups, it can be found that these target gene mutations and model riskScore together influence the patients’ survival (Figures 10C–F). Patients in the high risk group with the target gene mutation have the worst survival.

### Dynamic Nomogram and Multiple Validations of Model Genes

**FIGURE 11:**
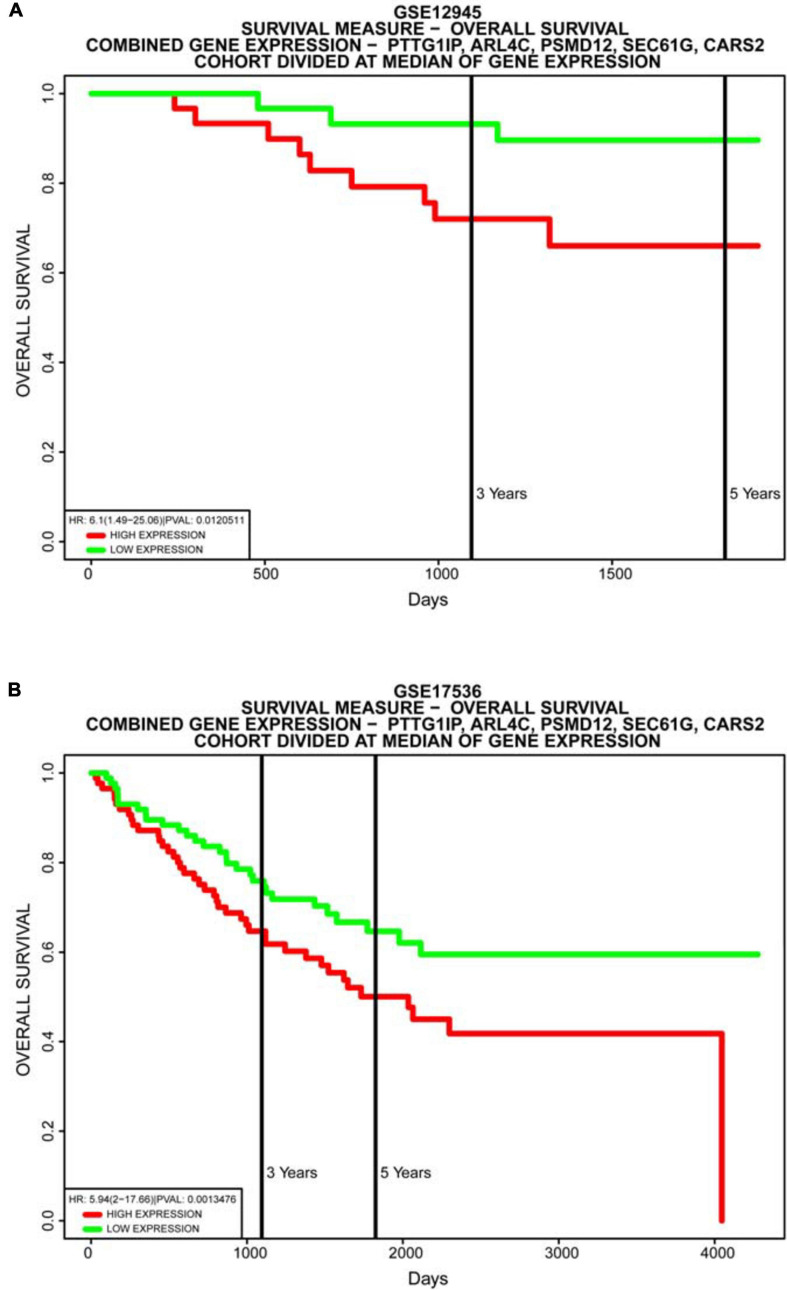
Database validation of the model. Multiple gene survival analysis, from the PROGgeneV2 online tool. **(A)** GSE12945; **(B)** GSE17536. They all successfully validate the accuracy of our model, with *P* < 0.05.

**FIGURE 12:**
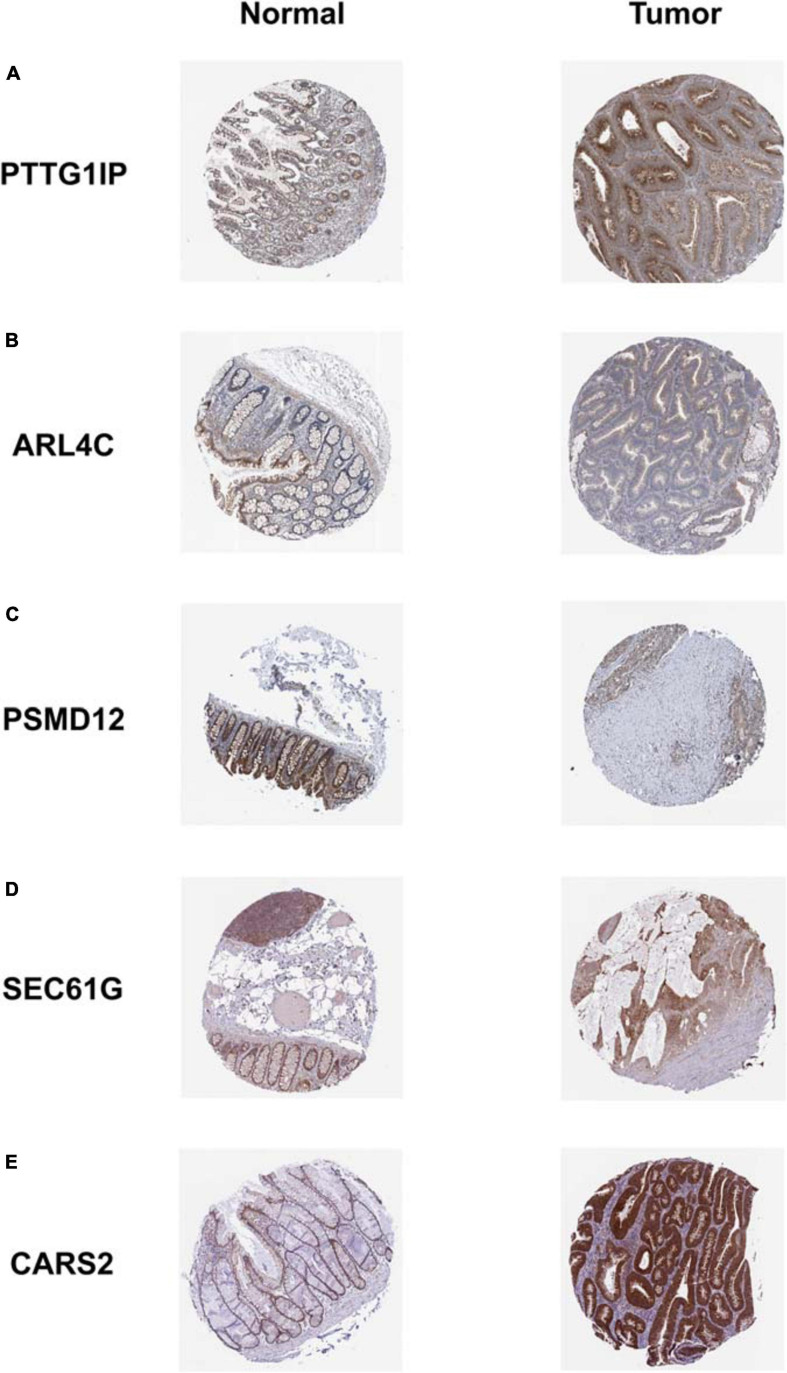
Immunohistochemical staining results of model genes. The expression trend of each model gene in tissues is basically consistent with the corresponding risk attribute. **(A)** PTTG1IP; **(B)** ARL4C; **(C)** PSMD12; **(D)** SEC61G; **(E)** CARS2.

The dynamic nomograph App online^1^ we developed enables clinicians to predict the survival rate of patients accurately. Our model is again validated by the PROGgeneV2 database (Figure 11). Immunohistochemical results downloaded from the HPA database display that the distribution of model genes in tissue is basically consistent with the risk attributes of model genes (Figure 12).

## Discussion

Exploration of hypoxia prognosis influence can reveal the role of hypoxia-related genes and pathways in the occurrence and development of COAD, and assist clinicians in judging the patients’ prognosis at the same time accurately. In this study, we applied a variety of bioinformatics methods to identify hypoxia genes related to prognosis through the comprehensive analysis of hypoxia characteristics and clinical information from COAD patients, and constructed an accurate hypoxia prognosis model successfully. In addition, we also discussed the relationship between hypoxia and immunity, and the effect of mutation on survival in COAD, which enriched the research level. In the end, the dynamic nomograph App online is convenient for direct clinical application, and the multiple validations of the model genes further enhance the preciseness.

In recent years, mature whole genome sequencing technology has provided great convenience for biological big data mining and promoted the rapid development of precision medicine. Previous studies have performed hypoxia-related analyses for certain types of cancer, while constructing prognostic signatures, including hepatocellular carcinoma (Jiang et al., 2020), gastric cancer (Chen et al., 2020), and melanoma (Shou et al., 2020). In terms of COAD itself, many previous studies have also proposed transcriptome signatures associated with COAD prognosis through bioinformatics analysis (Chang et al., 2020; Di et al., 2020; Luo et al., 2020). Currently, there are several hypoxia-related signatures that can predict clinical outcomes of colorectal cancer patients (Lee et al., 2019; Zou et al., 2019; Yang et al., 2020). Compared to these literature, the highlights and originality of our research lie in the application of LASSO senior algorithm to remove redundant genes, exploring the relationship between hypoxia and immune microenvironment to enrich the research level, developing a dynamic nomograph App online for clinical transformation, as well as including the multiple validations by external databases to enhance reliability. Our study further explores hypoxia-related biomarkers as prognostic predictors and can be used to predict patient outcomes and develop new treatment strategies.

The hypoxia model genes we identified as independent prognostic factors for COAD has also been reported in other studies. ARL4C has been reported to be highly expressed in metastatic colorectal cancer and is associated with poor prognosis. A novel targeted nucleic acid drug targeting ARL4C inhibits the expression of ARL4C in colorectal cancer cells, and ultimately reduces the proliferation and migration of cancer cells (Harada et al., 2019). Although there is currently no literature supporting the direct correlation between PTTG1IP and COAD, as a classical proto-oncogene, PTTG1IP has a significant tendency of overexpression and promotes cancer cell growth in other types of cancer, which is closely associated with poor clinical outcomes (including recurrence, metastasis and lower survival rate) in patients (Read et al., 2011, 2014; Wang et al., 2014). Similarly, although our preliminary analysis suggests that PSMD12 is negatively correlated with the progression of COAD, cytological experiments show that PSMD12 promotes the growth of breast cancer by inhibiting the expression of pro-apoptotic genes, which is expected to become a potential molecular target for prognosis and treatment (Du et al., 2020). No other studies have confirmed the relationship between SEC61G, CARS2, and cancer, so it is worth further in-depth study.

The result of ssGSEA analysis indicates that the overall hypoxia degree is positively correlated with the immune infiltration level in COAD patients. GSEA enrichment analysis suggests that the higher the hypoxia degree, the stronger the immunosuppressive activity, such as dendritic cell apoptotic process, negative regulation of alpha–beta T cell proliferation and T cell tolerance induction. This finding may provide a possible explanation for the lower survival rate in COAD patients with higher hypoxia level. Currently, it has been reviewed that hypoxia induces immunosuppressive process by affecting immune cell transport and angiogenesis in the tumor microenvironment, and provides a basis for clinical monitoring (Augustin et al., 2020; Pietrobon and Marincola, 2021). More specifically, potential mechanisms by which HIF-1 signaling in the tumor microenvironment promotes immune escape and leads to poor prognosis include: induction of immunosuppressor and immune checkpoint molecule expression, autophagy, exosome release, and novel immunogenic cell death (Vito et al., 2020; You et al., 2020). Studies on COAD have shown that hypoxia promotes immunosuppression by inhibiting the differentiation of CD4^+^ effector T cells and enhancing the activity of regulatory T cells (Westendorf et al., 2017). Hypoxia up-regulates the expression of VISTA and mediates the inhibitory function of myeloid-derived suppressor cells in the tumor microenvironment, providing another possible mechanism of hypoxia in immune escape for colon cancer (Deng et al., 2019). It can be seen that for COAD, the hypoxia degree is closely related to immune activity and plays an important role in the occurrence and development of cancer. The study on immunosuppression mechanism is helpful to reveal the significance of relevant pathways and provide a target for improving the immunotherapy efficacy.

Another important finding is that mutations in common target genes further refine the survival rate of patients in the high and low risk groups, which becomes an important reference for providing precise treatment and accurate prognosis judgment in clinical practice. Among them, patients in the high risk group with the targeted gene mutation have the worst survival, suggesting that this group may be most in urgent need of targeted therapies or combination therapies. Mutations of these common target genes can lead to the occurrence and metastasis of tumors, and their genotypes are closely related to the efficacy of targeted drugs.

We believe that the final multiple validations enhance the strictness of our conclusion, and the development of dynamic nomograph App online shows the practicability of our research. In addition, our conclusion needs to be further validated by wet experiments to make it more convincing.

## Conclusion

In summary, our study established an accurate hypoxia-related prognostic model for COAD patients and performed multiple validations. In addition, the correlation between hypoxia degree and immune activity, as well as the in-depth exploration of common target gene mutations, are also innovative findings of our study. These conclusions provide fundamental insights into the molecular mechanisms and prognostic markers of COAD, and may be useful for the future clinical translational application.

## Data Availability Statement

The datasets presented in this study can be found in online repositories. The names of the repository/repositories and accession number(s) can be found in the article/Supplementary Material.

## Author Contributions

GH and QL conceived and designed the study. YZ, FY, XP, and XL performed the literature search, generated the figures and tables, and wrote the manuscript. NL, WZ, and MF supervised the study and reviewed the manuscript. All authors read and approved the final manuscript.

## Conflict of Interest

The authors declare that the research was conducted in the absence of any commercial or financial relationships that could be construed as a potential conflict of interest.

## Footnotes

1 https://u201310506.shinyapps.io/COAD-hypoxia-Dynamic_nomogram/
