# Supplementary figures and images for: Hypoxia Constructing the Prognostic Model of Colorectal Adenocarcinoma and Related to the Immune Microenvironment
Source: Front Cell Dev Biol. 2021 Apr 20;9:665364. doi: 10.3389/fcell.2021.665364 (PMC8093637; doi:10.3389/fcell.2021.665364)

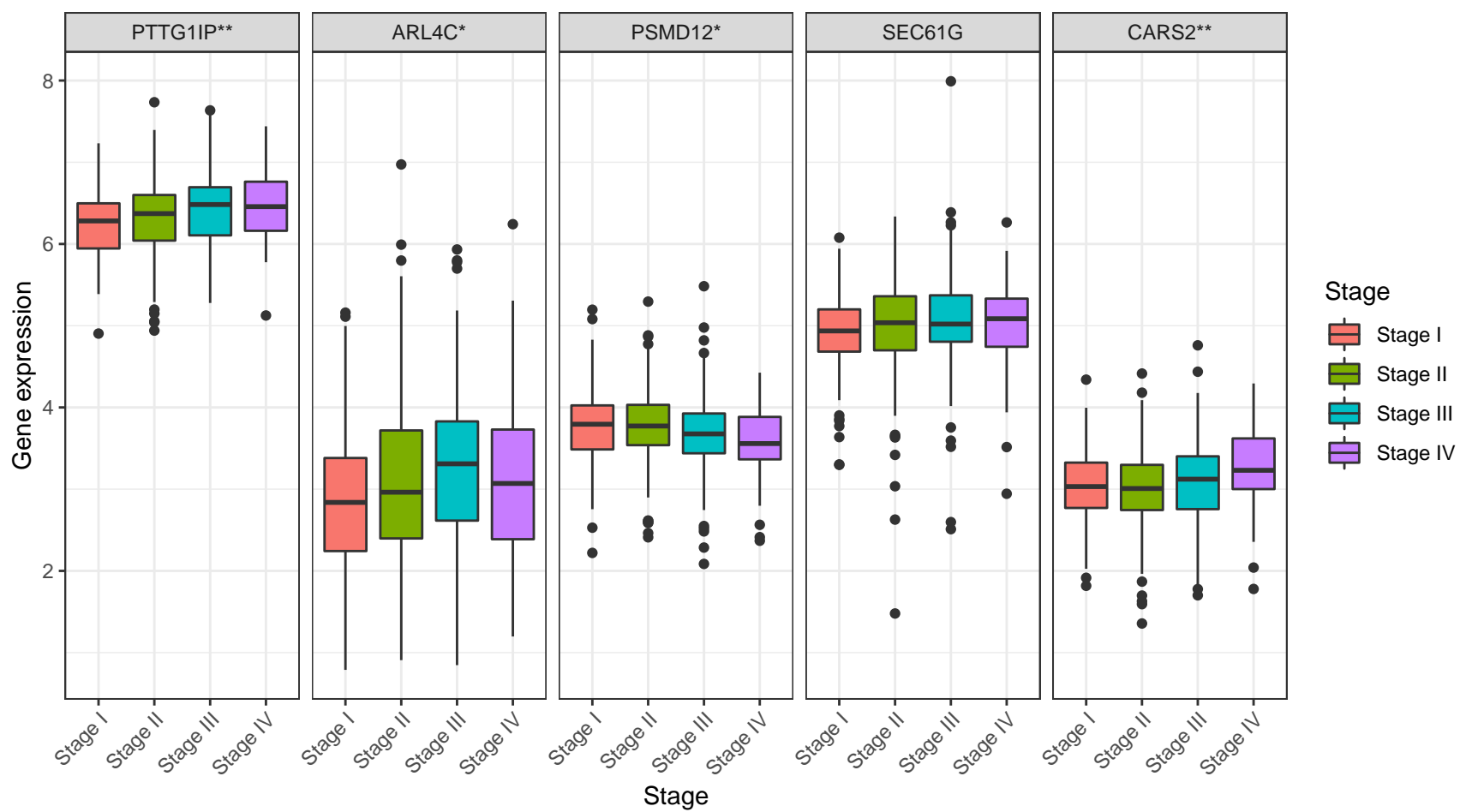

Supplement: Supplementary file 1 [file Data_Sheet_1.ZIP › Supplementary files/Supplementary file 10 - cliCor.pdf]

Gene expression

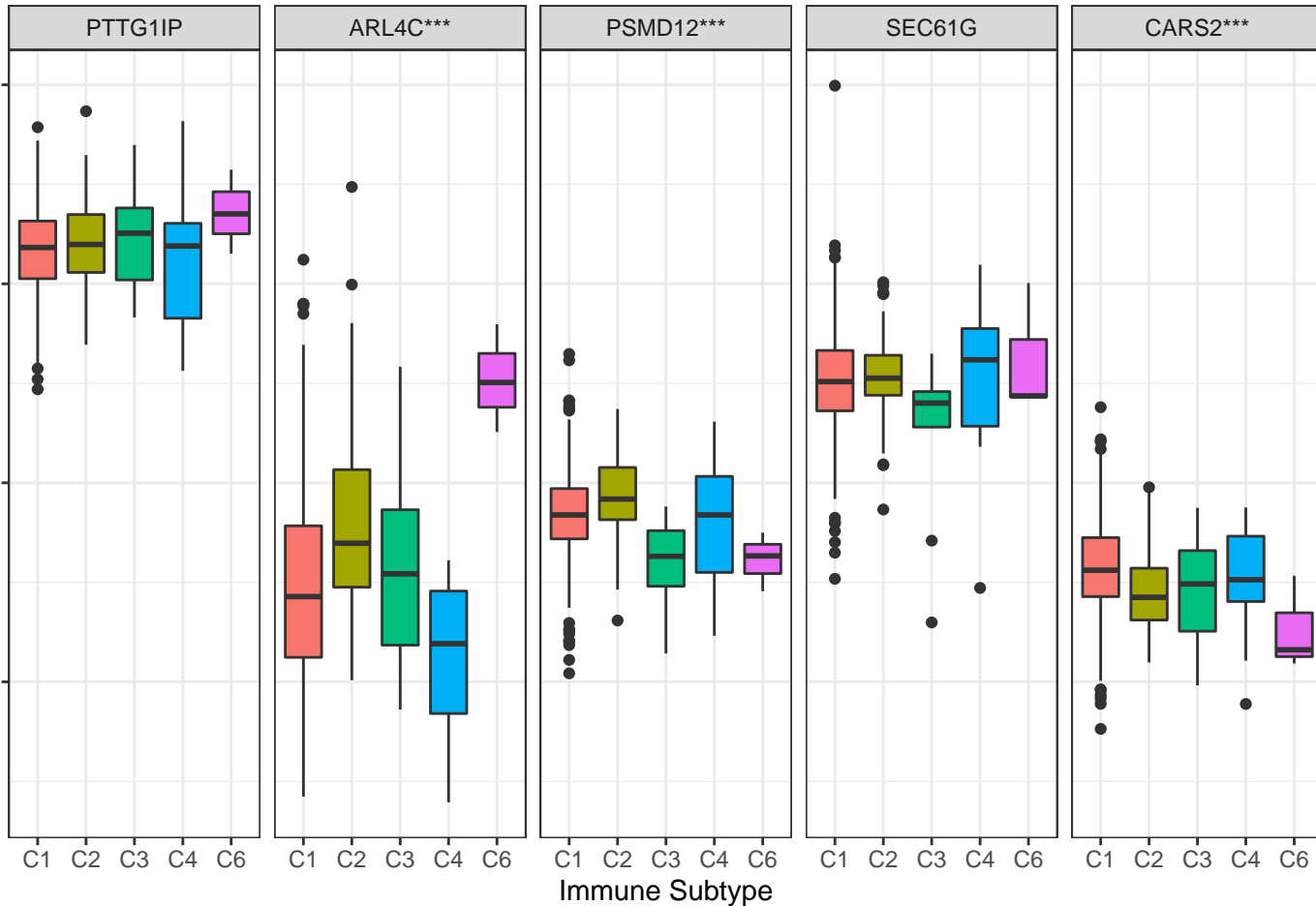

Immune Subtype

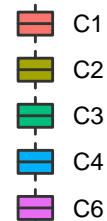

Supplement: Supplementary file 1 [file Data_Sheet_1.ZIP › Supplementary files/Supplementary file 11 - immuneType.pdf]

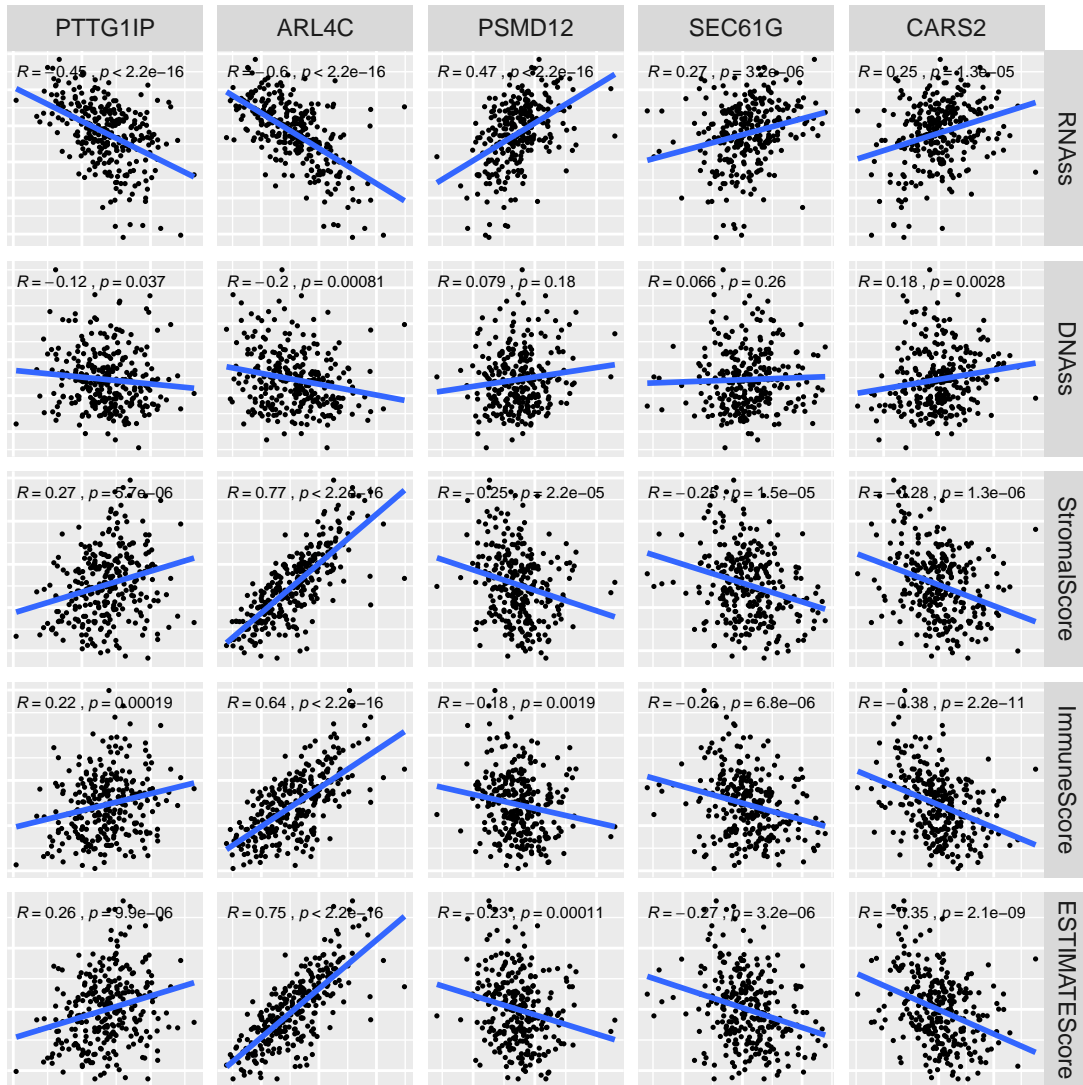

Gene expression

Supplement: Supplementary file 1 [file Data_Sheet_1.ZIP › Supplementary files/Supplementary file 12 - cor.pdf]
